# Yellow Fever in Charleston, S. C.

**Published:** 1849-12

**Authors:** 


*Yellow Fever in Charleston, S. C.*—The Editors of the Charleston Medical Journal and Review, state that yellow fever made its appearance in the city of Charleston August 5th, and has continued in an epidemic form. The number of deaths up to October 27 had been 109. As the cold weather had set in, it was anticipated that the disease would soon cease. This is the first time yellow fever has prevailed in Charleston in an epidemic form, since 1839.

